# Correction: Membrane expansion alleviates endoplasmic reticulum stress independently of the unfolded protein response

**DOI:** 10.1083/jcb.20090707402092021c

**Published:** 2021-02-15

**Authors:** Sebastian Schuck, William A. Prinz, Kurt S. Thorn, Christiane Voss, Peter Walter

Vol. 187, No. 4 | 10.1083/jcb.200907074 | November 9, 2009

Through a comment on PubPeer, the authors became aware that the Western blot in Fig. 7 A was assembled incorrectly. This mistake resulted in inadvertent duplication of the lanes showing Rtn1 in DTT-treated cells and mislabeling of the lanes showing Pgk1. The corrected figure panel is shown here. The interpretation of the data remains unchanged; that is, neither DTT treatment nor increased Ino2 activity affect Rtn1 abundance.

**Figure:**
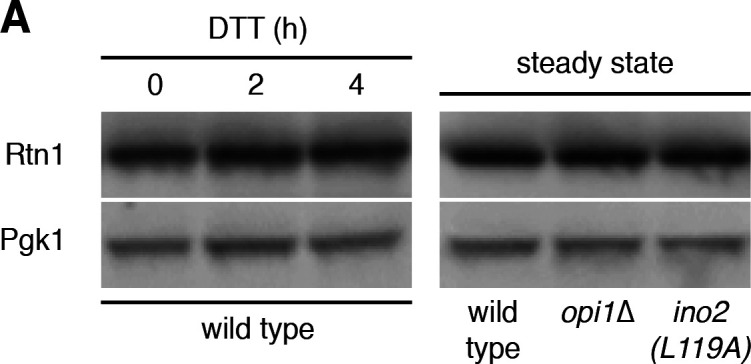


The authors apologize for their mistake and refer the reader to PubPeer for a more detailed description of how the mistake occurred: https://pubpeer.com/publications/63CD4A72F9C25B245D4A2A10FDDBC0.

